# Supplementary material for: Engineered probiotic Lactobacillus plantarum WCSF I for monitoring and treatment of Staphylococcus aureus infection
Source: Microbiol Spectr. 2023 Nov 1;11(6):e01829-23. doi: 10.1128/spectrum.01829-23 (PMC10848683; doi:10.1128/spectrum.01829-23)
Supplement: Fig. S1 to S10, Tables S1 — Specific instructions are indicated in the document. [file spectrum.01829-23-s0001.docx]

Supporting Information

**Engineered probiotic *Lactobacillus plantarum* WCSF I for monitoring and treatment of *Staphylococcus aureus* infection**

Haoran Li^1,2,^ Minjun Jia^1^, Qingsheng Qi^2^, Qian Wang^1^*

^1^National Glycoengineering Research Center, Shandong University, Qingdao 266237, P. R. China

^2^State Key Laboratory of Microbial Technology, Shandong University, Qingdao 266237, P. R. China

* To whom correspondence may be addressed.

Tel & Fax: +86-532-58631580, Email: [qiqi20011983@gmail.com](mailto:qiqi20011983@gmail.com); qiqingsheng@sdu.edu.cn

Supplementary Figures

Figure S1


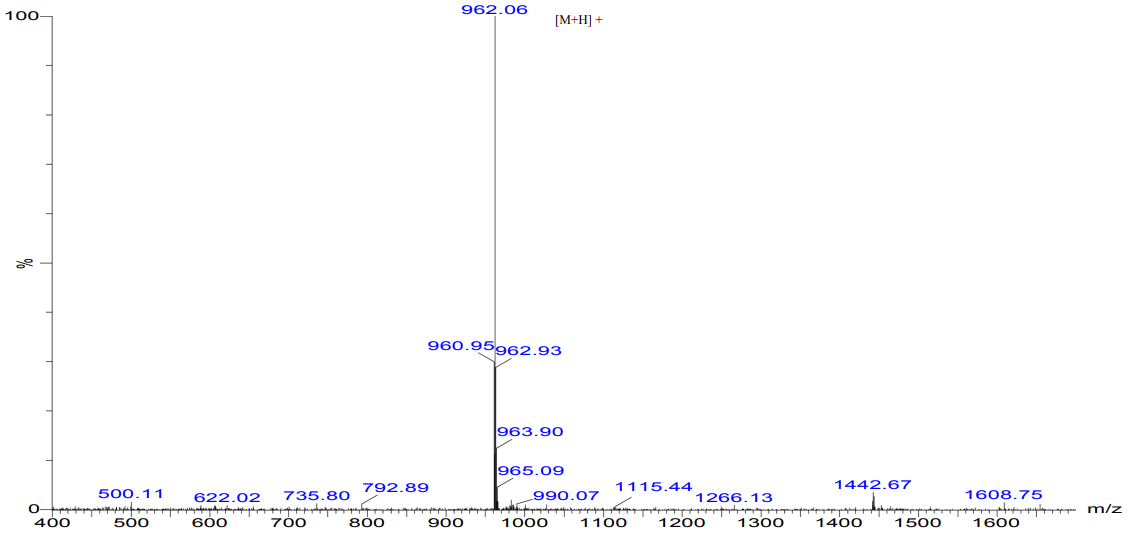


**Figure S1**. The liquid chromatography-mass spectrometry result for the *Staphylococcus aureus* supernatant.

Figure S2


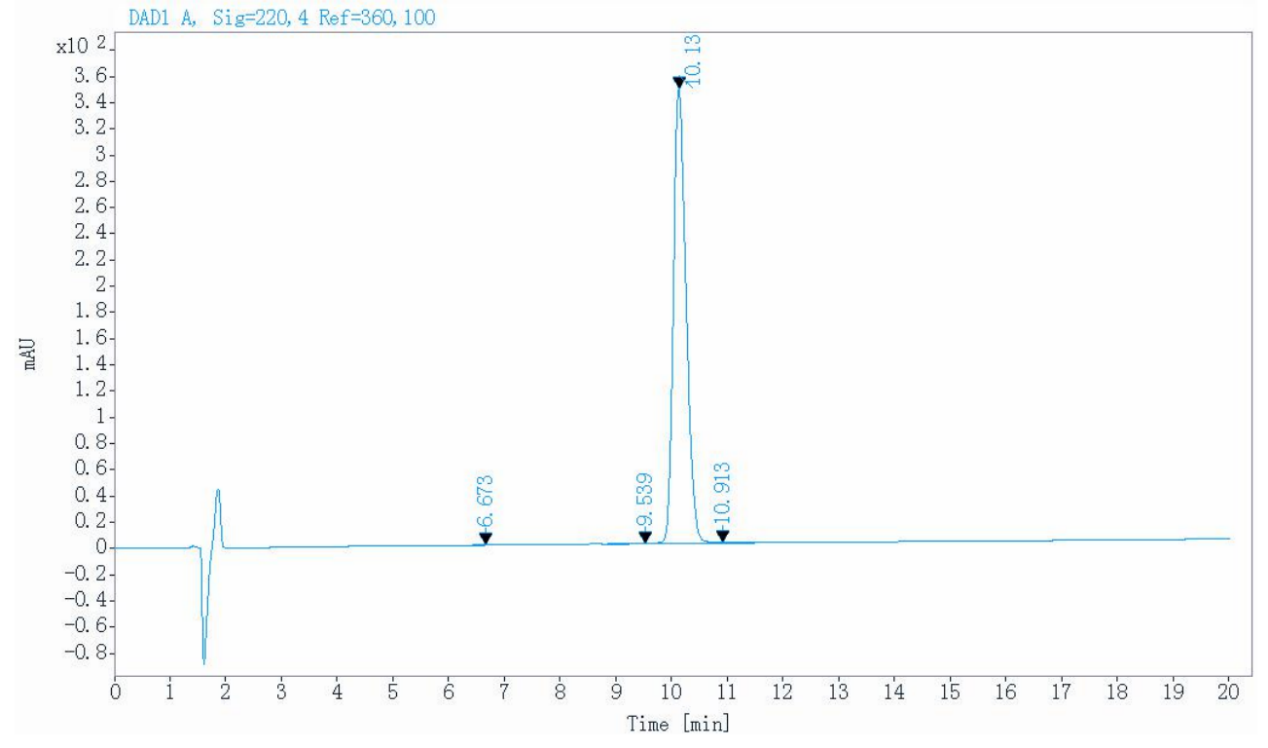


**Figure S2.** The high-performance liquid mass spectrometry result for synthetic AIP-Ⅰ.

Figure S3

**
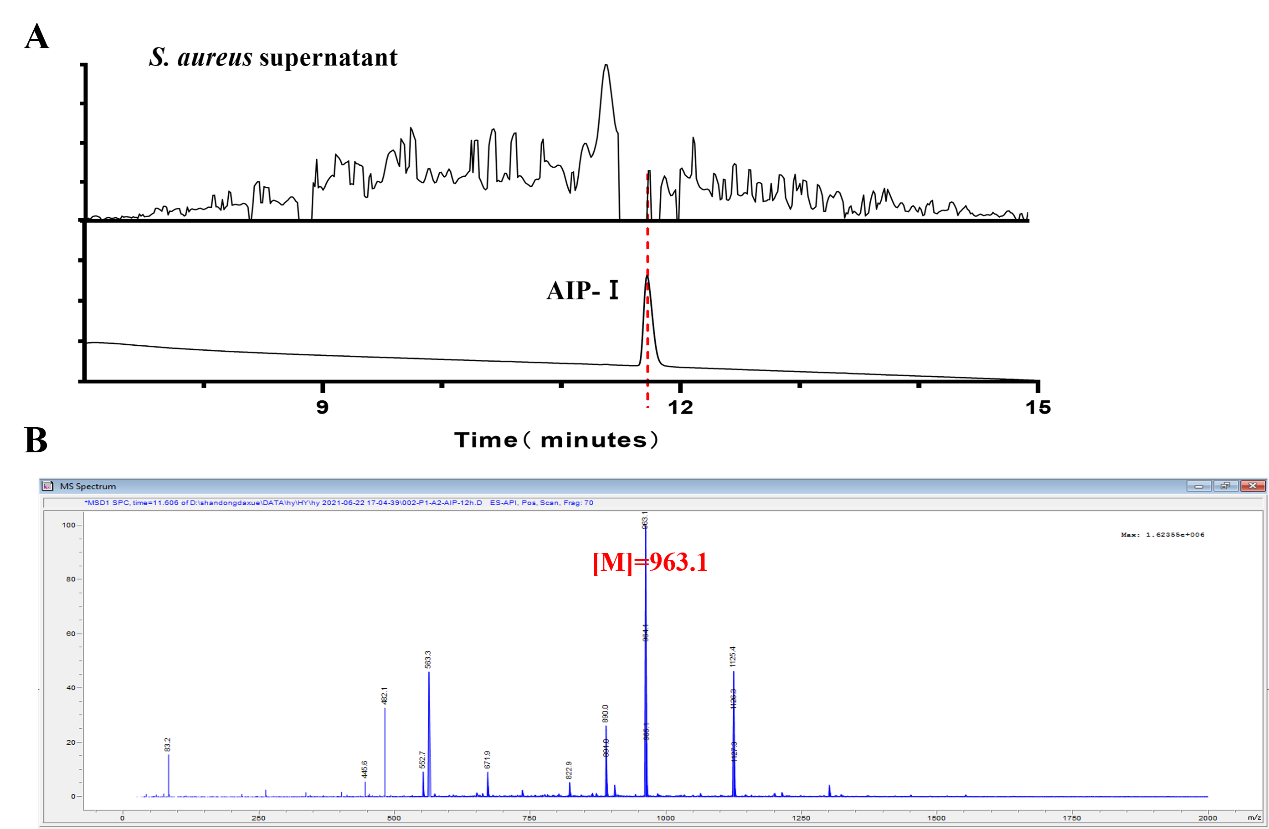
**

**Figure S3.** (A) HPLC chromatograms of AIP in *S.aureus* supernatant. (B) HPLC-MS detection of AIP in *S.aureus* supernatant.

Figure S4


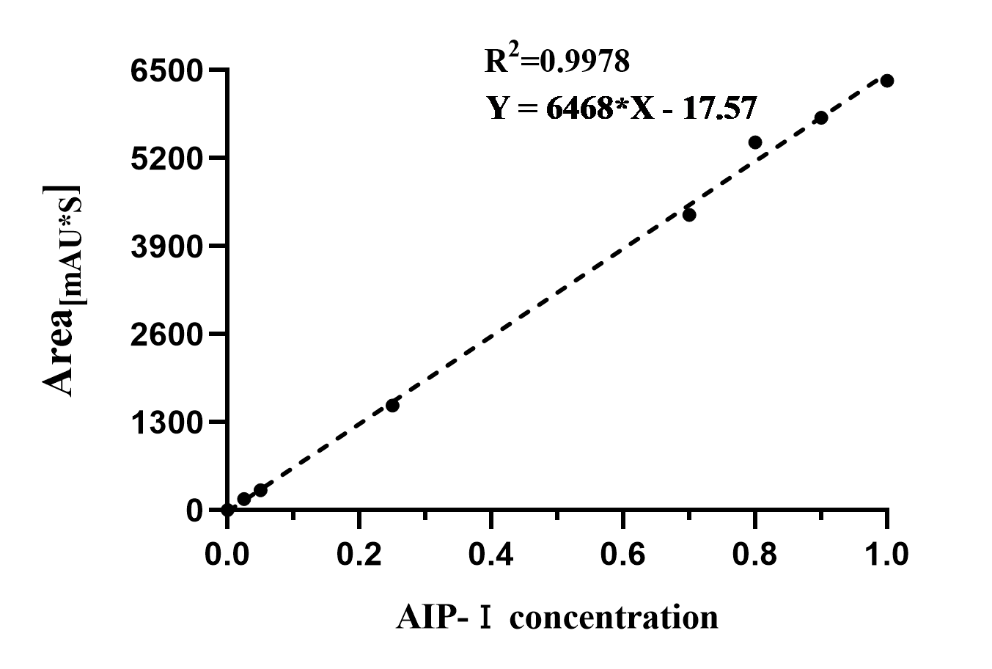


**Figure S4.** Standard curve of AIP-Ⅰ standard substance concentration and peak area.

Figure S5

**
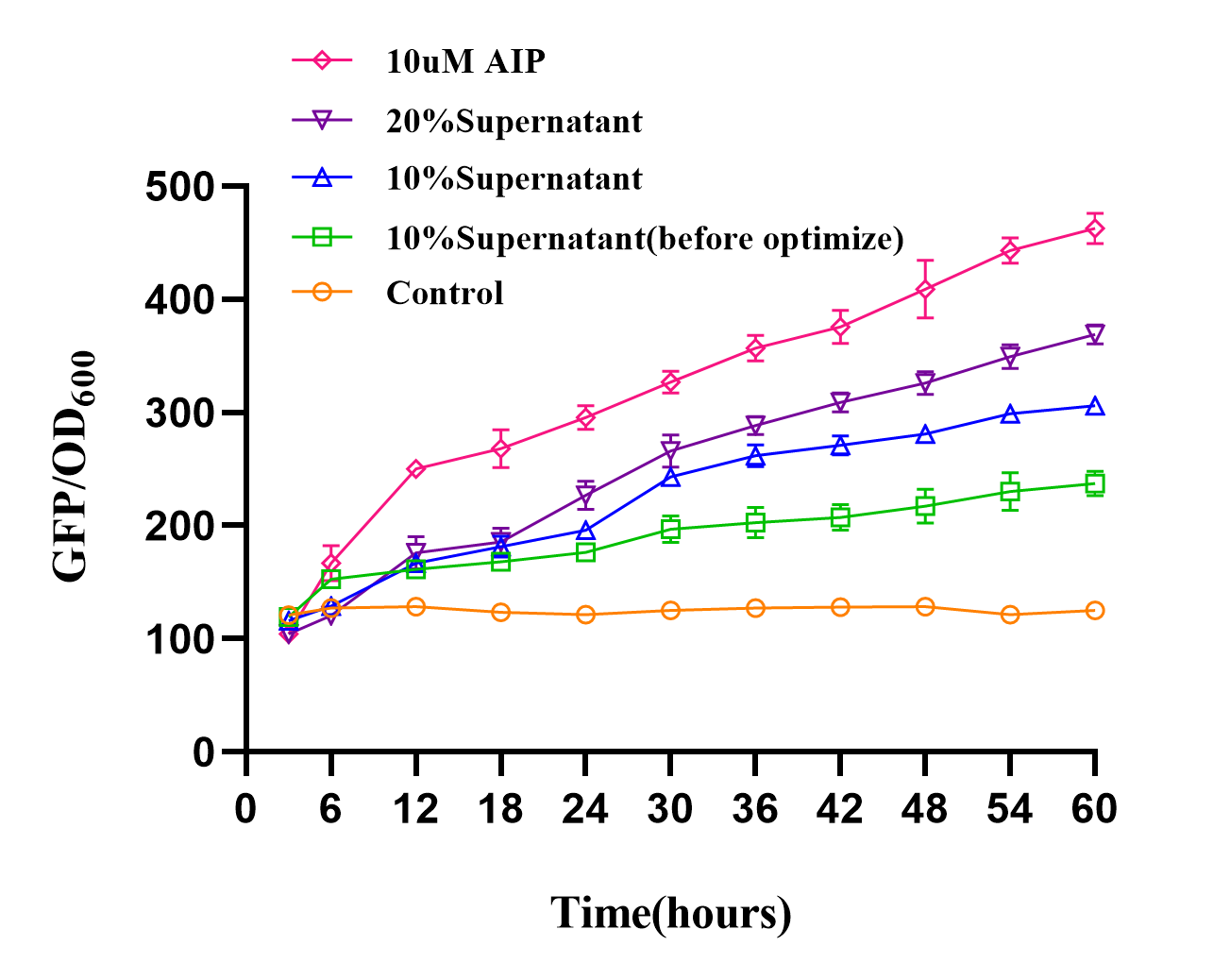
**

**Figure S5.** The time course of fluorescence intensity of AgrQS system using P_32_ promoter under different concentrations of AIP and *S. aureus* supernatant.

Figure S6

**
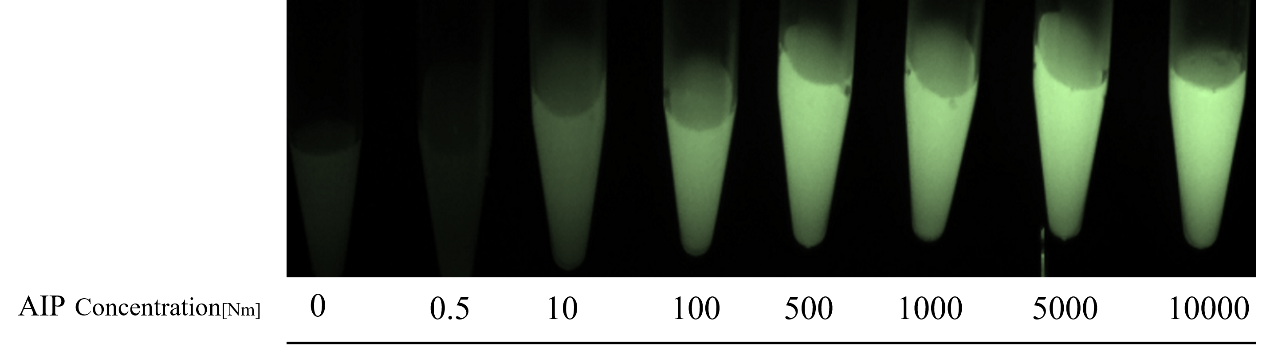
**

**Figure S6.** The luminescence of *Lactobacillus plantarum* was introduced to the P_32_-P_11_Mut-AgrQS system under the induction of different concentrations of AIP.


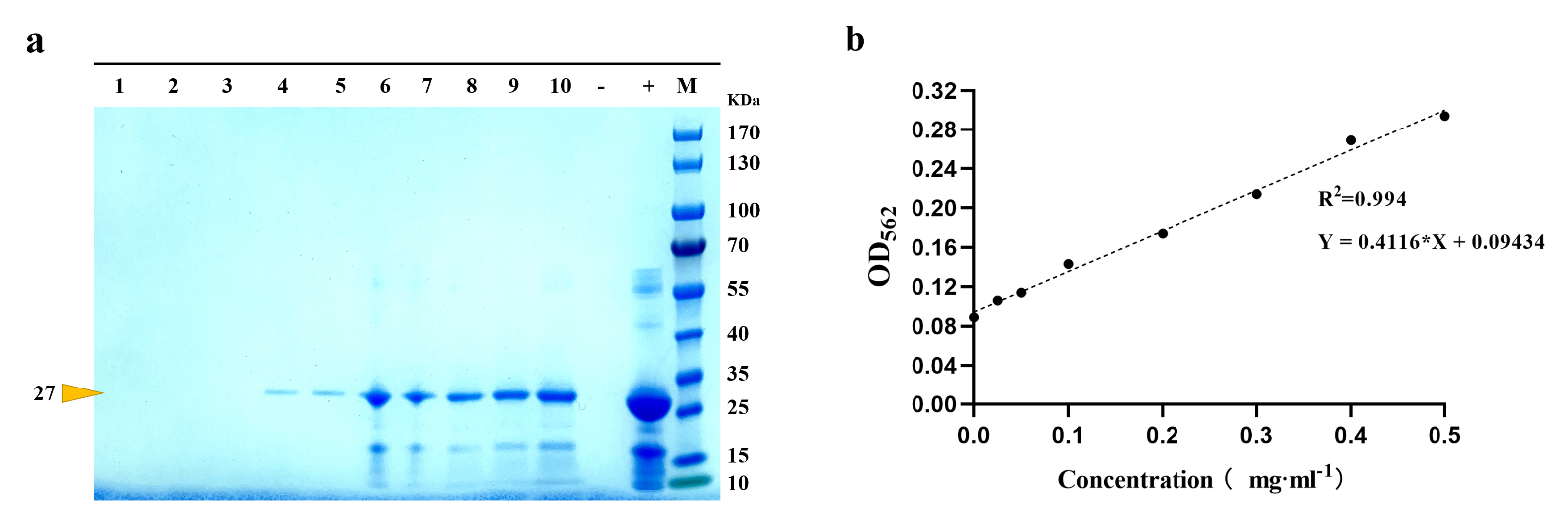
Figure S7

**Figure S7.** Engineered *L. plantarum* secretes lysostaphin enzyme. (a) Production of the lysostaphin enzyme using the AgrQS system at different AIP concentrations [Chanel 1: 0.5nM, Chanel 2: 1nM, Chanel 3: 10nM, Chanel 4: 50nM, Chanel 5: 100nM, Chanel 6: 250nM, Chanel 7: 500nM, Chanel 8: 1000nM, Chanel 9: 5μM, Chanel 10: 10μM]. The molecular weight of the lysostaphin enzyme was 27 KDa. (b) Lysostaphin concentration curve.

Figure S8


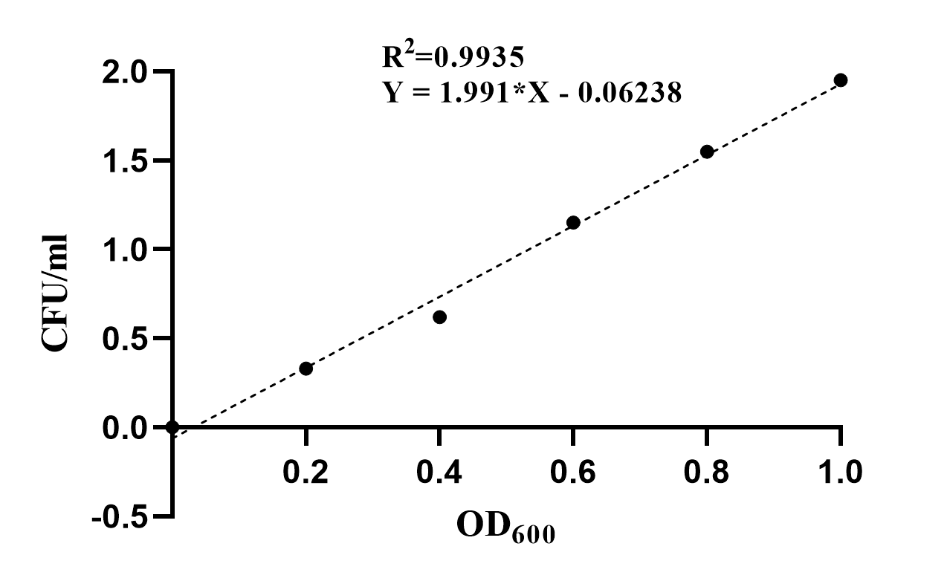


**Figure S8.** The corresponding curve of light absorption of *S. aureus* at 600 nm wavelength and the number of bacteria.

Figure S9


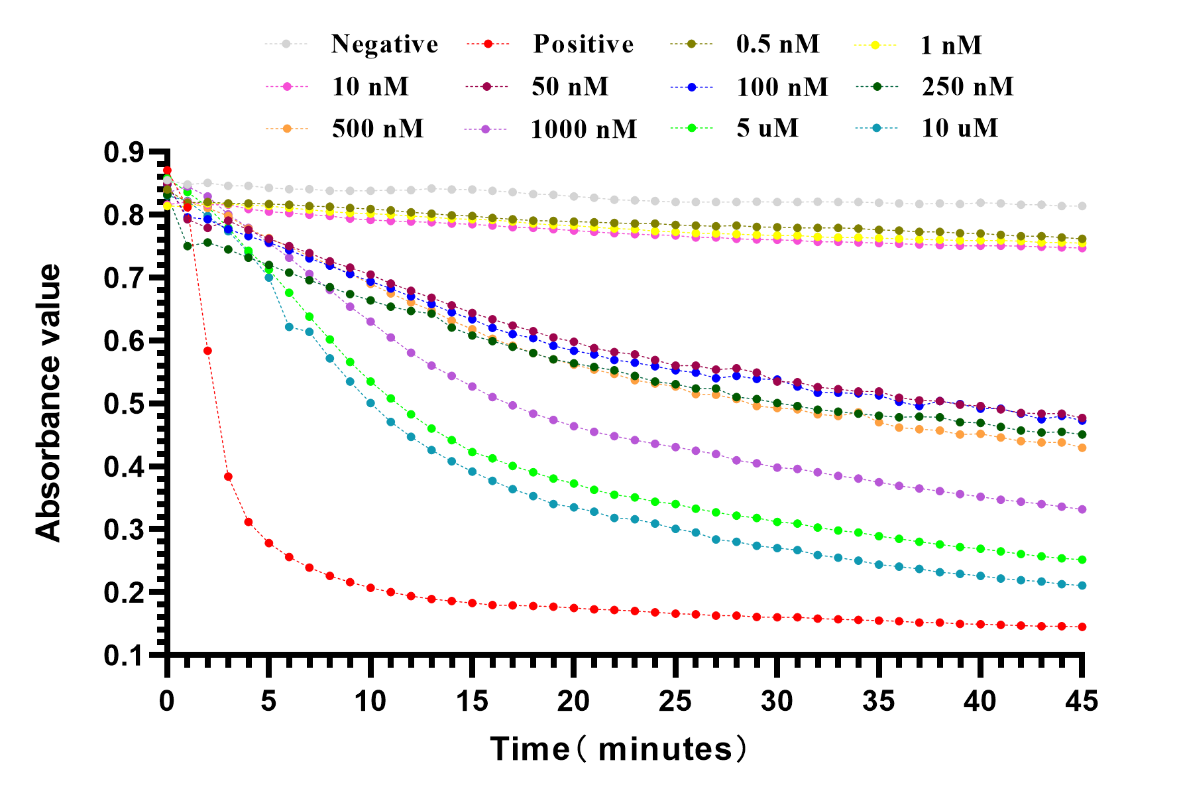


**Figure S9.** Growth kinetics of *S. aureus*. The growth of *S. aureus* cocultured with the *L. plantarum* supernatant.

Figure S10


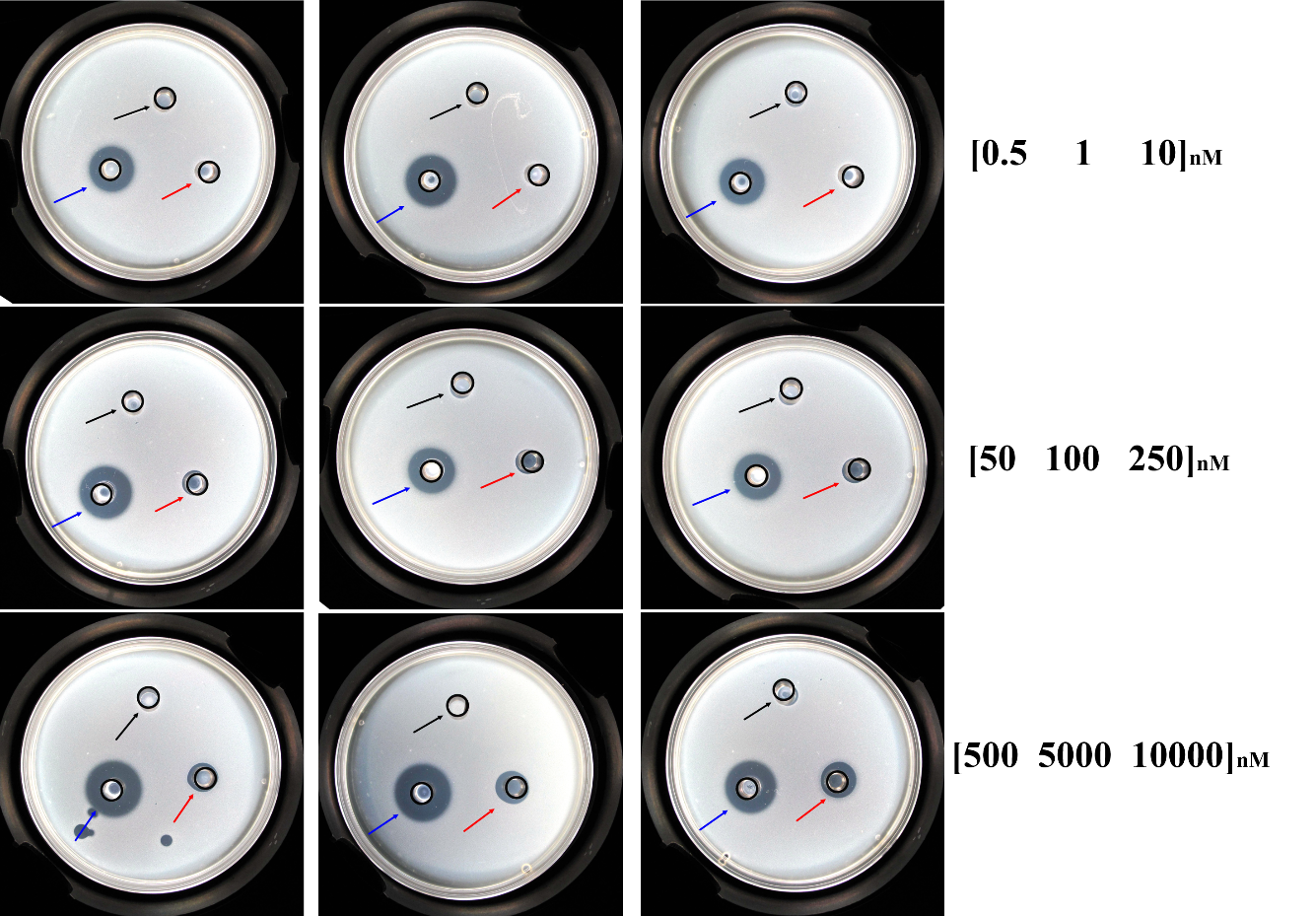


**Figure S10.** *S. aureus* tablet bacteriostatic experiment. After adding different concentrations of the AIP-induced *L. plantarum* supernatant to the LB plate inoculated with *S. aureus*, the bacteriostatic status of *S. aureus* was observed. (Added a phosphate buffer solution where the black arrow is pointing; added lysostaphin where the blue arrow is pointing; and added the *L. plantarum* supernatant after AIP induction where the red arrow is pointing.)

Supplementary Tables

**Table S1.** The core sequences of the P_3_, P_23_, and P_11_ promoters.

| Pro. | The core sequence |
| --- | --- |
| P_3_ | AAATTTACAGTTAAGAATAAAAAACGACTAGTTAAGAAAAATTGGAAAATAAATGCTTTTAGCATGTTTTAATATAACTAGA |
| P_23_ | TGATGACAAAAAGAGAAAATTTTGATAAAATAGTCTTAGA |
| P_11_ | ACTTGCAACCGTTTTCTATTTGTGCTATACTAAGCTCATAA |

The red words label represents the AgrA binding sequence. The blue words label represents the -35 box. The yellow words represent the -10 box.
